# Reply: Premature ovarian insufficiency and the risk of breast cancer

**DOI:** 10.1093/hropen/hoaf018

**Published:** 2025-04-09

**Authors:** Nick Panay, Nathalie Vermeulen, Richard A Anderson, Amanda J Vincent

**Affiliations:** Queen Charlotte’s and Chelsea Hospital, Imperial College London, London, UK; ESHRE Central Office, Strombeek-Bever, Belgium; Centre for Reproductive Health, Institute for Regeneration and Repair, University of Edinburgh, Edinburgh, UK; Monash Centre for Health Research and Implementation (MCHRI), Monash University, Clayton, Australia

Sir,

We read with interest the letter to the Editor ‘Premature Ovarian Insufficiency and the Risk of Breast Cancer’ by Coelingh Bennink *et al.* (2025) as well as the narrative review referred to (Coelingh Bennink *et al.,* 2023). However, they do not accurately reflect how this important topic is addressed in the recently published premature ovarian insufficiency (POI) guideline (https://www.eshre.eu/Guidelines-and-Legal/Guidelines/Premature-ovarian-insufficiency), and they propose a treatment approach that has significant safety concerns.

The authors raise the concern that the POI guideline ‘*does not address the decreased risk of breast cancer in women with POI*’. Although the summary guideline publications (Panay *et al.*, 2024a, 2024b, 2025) do not address this issue in detail, the section of the full guideline ‘Risk of breast cancer in women with POI’ does address this complex topic, citing the same publications, which indicate a 30–40% lower risk of breast cancer in women with menopause before age 40 years (Collaborative Group on Hormonal Factors in Breast Cancer, 2012; Wu *et al.*, 2014) and in those with POI secondary to risk-reducing bilateral oophorectomy for *BRCA1/2* gene carriers (Rebbeck *et al.*, 2009). However, a 2024 study of a cohort of 613 Polish women with POI, not cited by Coelingh Bennink *et al.* (2025) but included in the POI guideline, indicated a 2-fold increased risk of breast cancer with causal and candidate genes identified (Allen-Brady *et al.*, 2024). Thus, our understanding of the risk of breast cancer in this population is incomplete, with potential interacting factors including underlying genetic make-up, other reproductive and lifestyle factors, and hormone therapy.

The guideline systematic search focused on women with POI and, thus, studies of women with early menopause (Monninkhof *et al.*, 1999) or related to menstruation (Coelingh Bennink *et al.*, 2023) may not have been captured.

The guideline acknowledges the ‘considerable debate on the effect of different progestogens on the risk of breast cancer’ (Stahlberg *et al.*, 2004; Seeger and Mueck, 2008). In theory, progesterone has a less proliferative and a more apoptotic effect than androgenic progestogens. However, the evidence is largely observational and relates to women with usual age of menopause; as noted in the guideline, there are no data specific to POI (Schneider *et al.*, 2009; Vinogradova *et al.*, 2020).

We note that ‘these differing agonist and antagonist effects contribute to the variable adverse effects profile (for example, breast cancer or venous thromboembolism [VTE]) and this should be considered when deciding on the hormone therapy (HT) regimen’ (Stanczyk *et al.*, 2013).

These points all support the conclusions that:

- ‘There are no data to indicate an increased risk of breast cancer in women with POI using HT compared to an age-matched premenopausal women.’
- ‘Given the recognised long-term risks of untreated POI, recommendations were formulated to reassure women with POI that the pros are likely to outweigh the cons to use HT in this context, despite the lack of good quality breast safety data’.

We strongly disagree with the authors’ recommendation, ‘*we propose to avoid the use of progestogens and treat POI patients with estrogens only, […] monitor vaginal bleeding and endometrial growth regularly and only prescribe progestin withdrawal when needed*’. The authors do not give sufficiently serious consideration to the long-established risk of endometrial hyperplasia and cancer with unopposed estrogen therapy in postmenopausal women. A Cochrane review and other publications report odds ratios ranging from 12 to 67 in studies using moderate- to high-dose unopposed estrogen compared to sequential combined hormone therapy over only 3 years duration (Furness *et al.*, 2012; Sjögren *et al.*, 2016). Of concern, endometrial hyperplasia was reported in 62% of women receiving unopposed moderate-dose estrogen therapy for 3 years compared with 1.7% of women receiving placebo therapy (Furness *et al.*, 2012).

The guideline recommends hormone therapy for women with POI from diagnosis until the usual age of menopause, often resulting in decades of use. Monitoring of vaginal bleeding and endometrial thickness would not be a feasible or reliable strategy for all women with POI (Manley *et al.*, 2024) on unopposed HT: this would, by design, increase interventions and result in patient and clinician anxiety.

Such a strategy might also be proposed for postmenopausal women taking HT, but nowhere has it been adopted or supported, and there is no evidence that it can adequately minimize the known risk of unopposed estrogen therapy (Manley *et al.*, 2024).

Guideline methodology requires guideline development group consensus regarding recommendations. These are the relevant recommendations to this topic (Panay *et al.*, 2024a, 2024b, 2025):

**Table:** 

HT is recommended for women with POI until the usual age of menopause for primary prevention to reduce the risk of morbidity and mortality, whether there are estrogen deficiency symptoms or not.	STRONG	**⊕○○○**

Women with POI can be informed that there is no evidence that HT use increases their risk of breast cancer compared to women of the same age without POI.	CONDITIONAL	**⊕⊕○○**

A progestogen should be given in combination with estrogen therapy to all women with an intact uterus to prevent endometrial hyperplasia/cancer.	STRONG	**⊕⊕○○**

While feedback and opinion are part of the guideline process and actively requested during the stakeholder review, such feedback should be based on substantial evidence and devoid of commercial conflicts of interest. Whilst we welcome new studies that will increase the safety and efficacy of treatment for the diverse health concerns that women with POI face, our considered view is that there is no need for adaptation of the POI guideline or recommendations in the light of the arguments proposed by Coelingh Bennink *et al.* (2025).
